# Supplementary material for: RuvC uses dynamic probing of the Holliday junction to achieve sequence specificity and efficient resolution
Source: Nat Commun. 2019 Sep 10;10:4102. doi: 10.1038/s41467-019-11900-8 (PMC6736871; doi:10.1038/s41467-019-11900-8)
Supplement: Supplementary file 1 — Supplementary Information [file 41467_2019_11900_MOESM1_ESM.pdf]

SUPPLEMENTARY INFORMATION

RuvC uses dynamic probing of the Holliday junction to achieve sequence specificity and efficient resolution

Karolina Maria Górecka,[1, &] Miroslav Krepl,[2, &, *] Aleksandra Szlachcic,[1] Jarosław Poznański,[3] Jiří Šponer,[2, 4] and Marcin Nowotny[1, *]

[1] Laboratory of Protein Structure, International Institute of Molecular and Cell Biology, 4 Trojdena St., 02-109 Warsaw, Poland

[2] Institute of Biophysics of the Czech Academy of Sciences, 135 Kralovopolska St., 612 65 Brno, Czech Republic

[3] Institute of Biochemistry and Biophysics Polish Academy of Sciences, 5a Pawinskiego St., 02-106 Warsaw, Poland

[4] Regional Centre of Advanced Technologies and Materials, Faculty of Science, Palacky University Olomouc, 27 Slechtitelu St., 771 46 Olomouc, Czech Republic

[&] These authors contributed equally to this work.

* To whom correspondence should be addressed. e-mail: mnowotny@iimcb.gov.pl, krepl@ibp.cz

Supplementary Notes

Comments on the utilized force-field parameters and the MD simulation protocol

We used the ff12SB [1] and OL15 [2] force fields for protein and DNA description, respectively. Both of these force fields are reparametrized versions of the Cornell *et al.* force-field parametrization from 1995 [3]. This original parametrization successfully described electrostatic interactions by fitting atomic partial charges to reproduce the electrostatic potential of individual nucleotides and amino acids. This allowed stable simulations of proteins and nucleic acids on a picosecond to nanosecond simulation timescale. However, issues observed on longer simulation timescales were subsequently reported in the literature, prompting development of several new reparametrizations of dihedral potentials and also separating the development of protein and nucleic acids force-fields.

Thus, the protein force field utilized in this work, in addition to the basic Cornell et al. parametrization, includes the SB modification from 2006 [4]. This modification improved the secondary structure balance and dynamics by introducing new backbone dihedral parameters, but it did not deal with side-chain rotamer behavior. The behavior of the side-chains was addressed in 2015 by reparametrization of all amino acid side chain dihedral potentials, resulting in ff12SB and ff14SB versions of the protein force field [1]. Note that ff12SB was utilized in this work instead of ff14SB due to the latter one's controversial implementation of the tyrosine and phenylalanine dihedral potentials [5].

The description of DNA by the force field is inherently more difficult than for proteins due to the highly correlated conformations of the sugar-phosphate backbone and the polyanionic character of nucleic acids [6]. The OL15 variant of the DNA force field [2] utilized in this work represents developments spanning almost a decade which resulted into systematic reparametrization of all backbone dihedral potentials of DNA nucleotides and of their N-glycosidic χ potentials. The bsc0 force-field version [7] from 2007 reparametrized alpha and gamma dihedrals which was essential for

long-term stabilization of DNA double helices in simulations longer than ca. 10 ns. This was followed by χOL4 reparametrization [8] of the N-glycosidic dihedral potential in 2012 and by εζOL1 reparametrization [9] of epsilon and zeta potentials in 2013. These modifications improve description of both canonical and non-canonical DNAs and correct the description of the B-DNA helical twist. Finally, the βOL1 reparametrization from [2] 2015 improved the beta dihedral potential. The final DNA force field utilized in this work (abbreviated as OL15) is a complete reparametrization of the DNA dihedral potentials of the original Cornell et al. force field and is the currently recommended default AMBER force field for DNA simulations.

The MD simulation protocol utilized in our study is highly similar to the one utilized in the past simulation studies of nucleic acid and protein/nucleic acids systems [10,11]. Namely, after the initial system building in tleap module of AMBER 16 (see the main text), all systems were minimized and equilibrated by sander.MPI in a series of alternating minimization and equilibration steps with an incrementally decreasing positional restraint applied to the RuvC and the DNA (i.e. the solute). In the initial minimization step, the systems were subjected to 500 integration steps of deepest descent and conjugated gradient, respectively, for a total of 1000 integration steps of minimization with a positional restraint of 25 kcal/mol/Å^2 placed on the solute. The same minimization protocol was applied in subsequent minimization runs except of the lower positional restraint applied at each step. After the first minimization, the systems were gradually heated up from 100 K to 300 K in a 100-ps-long NVT (constant volume) simulation along with a positional restraint of 25 kcal/mol/Å^2 placed on the solute. We used Berendsen weak-coupling with its default coupling value of 1 ps to regulate the temperature in this simulation. After this step, we performed a minimization with a positional restraint of 5 kcal/mol/Å^2 placed on the solute. This was followed by 50-ps-long NPT (constant pressure) equilibration simulation with the same positional restraint of 5 kcal/mol/Å^2 placed on the solute. From this point on, we used Berendsen weak-coupling thermostat and barostat to regulate the temperature and pressure, respectively. Coupling value of 0.2 ps was used for both.

Afterwards, we repeated the cycles of minimizations and equilibrations with incrementally lowering positional restrain of 4, 3, 2, 1, and 0.5 kcal/mol/$Å^2$, respectively, placed on the solute. At the end, the systems equilibrated in this fashion were used to start production MD simulations using the pmemd.cuda [12]. Particle mesh Ewald [13] with the periodic boundary conditions applied was used to describe the electrostatic interactions in all simulations. We used a cut-off distance of 9 Å for the non-bonded Lennard-Jones interactions.

Initial MD simulation of RuvC-DNA complex

The initial MD simulation based on the new X-ray structure of RuvC-DNA complex revealed a stable protein-DNA interface. The H-bond interactions between the sugar-phosphate backbone of DNA and the Ile10, Thr11, Lys83, and Arg47 amino acids that were observed in the X-ray structure were fully maintained in the simulation (Supplementary Figure 2c). Thus, the initial simulation supported applicability of the computational methodology for the studied system.

Since the X-ray structure contains a non-cognate DNA sequence at both catalytic sites, all subsequent simulations were conducted with modified DNA containing cognate nucleotide sequences at both catalytic sites (Supplementary Table 2). Identical protein-DNA interface interactions were observed in those simulations and the behavior described below and in the main text was observed for both symmetrical parts of the system (Supplementary Figure 2).

Comments on the use of Mg^{2+} and K^+ ions in MD simulations of the RuvC-HJ complex

We performed sets of simulations in which either four Mg^{2+} ions were placed at their expected positions at catalytic sites of RuvC or in which no magnesium was included in the system. Interestingly, even in simulations that did not include Mg^{2+} ions (i.e., a necessary component of RuvC catalysis) [14], K^+ ions from the bulk filled the magnesium binding pockets in the catalytic centers. K^+ ions cannot replace Mg^{2+} ions in the actual enzymatic reaction, but experimental data show that they can occupy Mg^{2+}-binding sites at nuclease active sites. The structural substitutability of Mg^{2+} by

monovalent ions has also been demonstrated computationally [15] and experimentally [16] at the catalytic center of hepatitis delta virus ribozyme and in the crystal structure of RNase H1 [17]. Appropriate positions of the magnesium ions in simulations of the RuvC-DNA complex were determined based on high-resolution structure of the *Tt*-RuvC protein (PDB: 4EP4) [18] and structures of the RNase H domain which possess a structurally similar catalytic site. The positions of magnesium ions were further refined by use of distance restraints in a series of short MD simulations.

Enhanced sampling simulations reveal the formation of cross-junction Arg76/adenine/adenine/Arg76 stacking

At the end of the standard MD simulations we observed that the adenine base randomly fluctuated after disruption of the scissile T-A base pair. Nevertheless, we suspected that the unpaired adenosine could be capable of further conformational changes that were, however, unreachable within the timescale of standard MD simulations [6]. Therefore, we performed enhanced sampling REST2 simulations to further explore the conformational landscape of the HJ's branching point in the RuvC-DNA complex (see Methods).

The initial stages of the REST2 simulations revealed development that was identical to the standard MD simulations, including eventual disruption of the scissile T-A base pair and thermal fluctuations of the unbound adenine. However, in later stages, the adenine eventually sampled the central region of the HJ. A single adenine was unstable in this position. However, when the adenines from the scissile T-A base pairs from both catalytic sites drifted into this region simultaneously, we observed immediate formation of what we term a "cross-junction stack" (Supplementary Figure 4). The formation of this cross-junction stack essentially prevented any re-formations of the T-A scissile base pairs within our simulation timescale. This adenine-adenine stack was further stabilized by the Arg76 side-chains that would either directly accompany the adenines during their movement toward the center of the junction or approach the existing adenine/adenine stack and themselves stack with the bases. In other words, the REST2 simulations showed that after extensive thermal fluctuations,

the two adenines from the disrupted scissile T-A base pairs can form a very stable base stack that is supported by stabilizing protein-DNA interactions. This development was consistently observed both across the replica space and in multiple demuxed trajectories of all the attempted REST2 simulations of RuvC-DNA containing the cognate sequence.

In conclusion, the REST2 simulations predicted conformational changes in the RuvC-DNA complex, in which the Arg76/adenine/adenine/Arg76 cross-junction stack forms in the center of the junction (Supplementary Figure 4). We do not claim a full convergence within the current simulation timescale, which was still relatively short (Supplementary Table 2). In fact, total convergence is not reachable within comparable timescales even for simpler nucleic acid systems, such as RNA tetraloops [6]. Additionally, the force field may not be sufficiently accurate to describe the free-energy balance between the two states. Nevertheless, the trend toward formation of the cross-junction stack is clearly recognized and consistently observed in the data. Importantly, the cross-junction stack was also entirely stable in the standard MD simulation derived from an enhanced-sampling simulation snapshot (Supplementary Table 2) containing the cross-junction stack (Supplementary Figure 4), thus confirming that it is a major substate in the free-energy landscape of the studied system with a sufficient lifetime once it forms.

Phe73 may be involved in recognition of the second base pair of the consensus sequence

The second residue of the A/TTT↓C/G consensus sequence in the RuvC-HJ complex is located at the branch point in the non-cleaved arms B1 and B2 (Figure 1, 6a). The RuvC preference for T-A over A-T in the base pair in this position was previously established experimentally [19-21]. The simulations provided some insights into possible origins of the observed sequence preference, although less clear than for the scissile base pair. The reason for this could be that base pair preference is related to processes that occur during binding of the substrate rather than during equilibrium dynamics of the fully bound complex. We extensively sampled the latter in our simulations, but the former would require simulations of binding-unbinding events. Some such

calculations could be possible in principle, but we doubt that they could be achieved in a reliable manner. Because we did not observe any binding-unbinding events of the DNA substrate within our simulation timescale, the origin of the sequence-dependence of the preceding base pair could be obscured.

Nevertheless, we found that the Phe73 side-chain, which is highly evolutionarily conserved in RuvC, directly contacts the second consensus base pair in the simulations. Specifically, in simulations that contained the preceding T-A base pair, we observed that the Phe73 side-chain interacted with the base-sugar edge of the adenine. Comparisons with simulations in which we changed the preceding T-A base pair to A-T or C-G showed that the specific interaction between Phe73 and the base-sugar edge was best maintained with the adenine. The reason for this is that all of the other bases contain bulky exocyclic groups at their base-sugar edges (e.g., O2 carbonyls in cytosine/thymine and N2 amino in guanine), which sterically clash with the Phe73 side-chain in simulations and weaken its interaction with the base-sugar edge. The preference of RuvC for a preceding T-A base pair could be attributable to its sequence-specific interaction with Phe73 (Supplementary Figure 9). The experimental verification of this assumption is hampered by the virtual lack of activity of *Tt*-RuvC with substitutions of Phe73 or its equivalents in other species [22,23].

Although not currently supported by simulations or experimental data, another hypothetical explanation could be formation of the adenine/adenine cross-junction stack that is analogous to the one that is observed for the scissile base pair (see above and Supplementary Figure 4). The involvement of adenines from the preceding base pair in the cross-junction stack was never observed in the simulations, but the formation of an asymmetrical cross-junction stack between the adenine from the scissile base pair and the adenine from the second base pair of the consensus sequence (Supplementary Figure 10) would be structurally possible. It could then also contribute to the acceleration of cleavage.

Simulation of free HJ DNA revealed large-scale dynamics near the branching point

The MD simulations of the RuvC protein-DNA complex revealed functionally significant dynamics of the substrate and suggested that the ability of various DNA substrates to undergo such dynamics could be a factor in determining the enzymatic specificity of RuvC (see main text). Therefore, we also explored the dynamics of HJ DNA in a free state for comparisons with the protein-DNA complex. The structure of free HJ DNA was prepared from the complex structure by removing the protein, obtaining an opened (tetrahedral) HJ structure. However, one of the challenges that had to be overcome was the fact that in the MD simulations, the opened HJ DNA rapidly converted to a so-called "closed state." In this conformation, stacking interactions form between base pairs in adjacent arms of the junction, and the internal dynamics of the junction are drastically reduced. Nevertheless, experiments have shown that the open-state conformation of the HJ as observed in the RuvC protein-DNA complex can also exist in free HJ DNA in solution. Furthermore, the open conformation is relevant for most enzymes that act upon HJ structures. After reviewing the simulation literature [24-27], we suspect that the absence of the open state in our MD simulations of free HJ DNA might be a simulation artifact that arises from the basic approximations that are used to describe simulation ions [6]. In our initial simulation attempts, the cations immediately surrounded the opened structure of free HJ DNA. By forming a set of phosphate-ion-phosphate interactions, they gradually forced it into a permanently closed state. The use of relatively small simulation box sizes may further exacerbate this problem, but we were unable to prevent this behavior by increasing the size of the simulation box or by limiting the number of ions within the system.

Eventually, we achieved a successful 500 ns simulation of opened HJ DNA through the total exclusion of explicit ions from the system. In such calculations, the overall zero net-charge of the system is maintained by uniform neutralizing plasma rather than by the addition of the corresponding number of explicit ions. In the past, this procedure was used to study potential late-stage folding intermediates of DNA and RNA quadruplexes [28]. In this simulation of free HJ DNA, we observed extensive dynamics at the junction point and several base pair re-pairing events within the

simulation timescale. Re-pairing events refer to rearrangements in which base pairs in two opposite arms of the junction are disrupted and then reconstituted in the other two arms (Supplementary Figure 13). This results in the extension of two arms by one base pair while the remaining two arms are shortened and corresponds to a naturally occurring and experimentally described branch migration process. This is only possible with a substrate that contains a homology region around the exchange point, such as with the cognate HJ DNA sequence that is recognized by RuvC.

The complete absence of explicit counter-ions limits the interpretability of this simulation, but it clearly shows that free, opened (tetrahedral) HJ DNA is extremely dynamic near the branching point. Our simulation results of the RuvC protein-DNA complex (main text) suggest that RuvC may have evolved to functionally exploit the substrate's extensive dynamics rather than suppress it entirely upon binding.

Supplementary figures and tables

Supplementary Figure 1

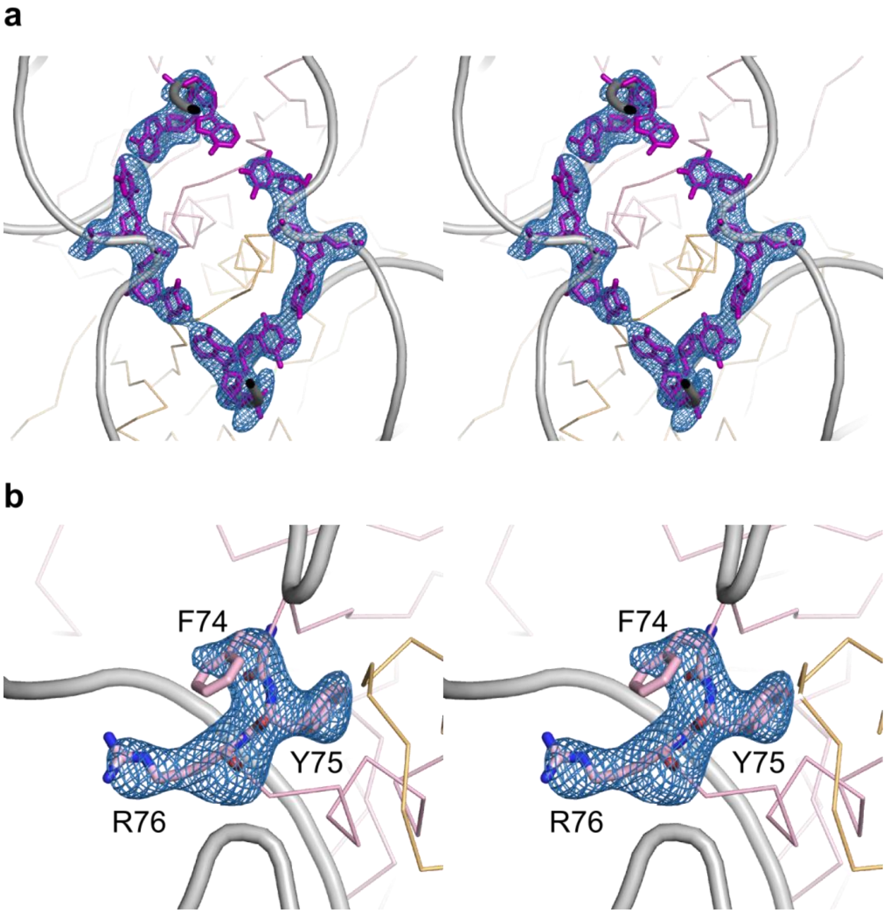

Supplementary Figure 1. Sample electron density maps (stereo view). Maps were calculated after the selected regions were omitted and refinement was performed with simulated annealing step to remove the model bias. (**a**) Close-up view of HJ exchange point with bases at the exchange point shown as sticks, DNA backbone is shown as gray cartoon and the protein is shown in wire representation. Fo-Fc omit map calculated for a model with nucleotides shown as sticks omitted is drawn as blue mesh and contoured at 3.5 σ . (**b**) Amino acid residues of the wedge element. Fo-Fc omit map calculated for a model with the three residues omitted is shown (contoured at 3.5 σ).

Supplementary Figure 2

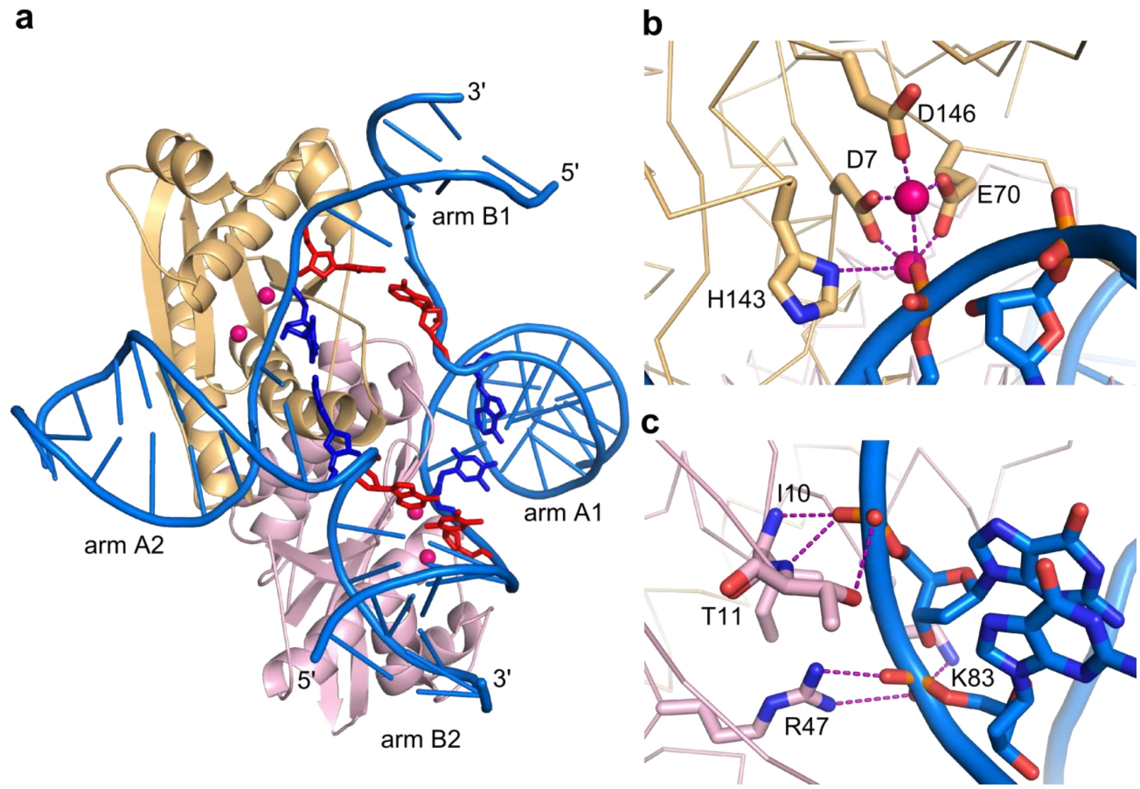

Supplementary Figure 2. Overall view of the simulated RuvC-DNA complex that contains the cognate DNA sequence. (**a**) Overall structure of the complex. The subunits of the RuvC dimer are shown in pink and light orange. The DNA is depicted as a blue ladder. Arms of the HJ and α-helix B are labeled. Magnesium ions at the active site are shown as pink spheres. (**b**) Close-up view of the active site. Residues that form the active site are shown as sticks and labeled. (**c**) Close-up view of the key contact between the protein and the cleaved arms of the HJ (A1 and A2).

Supplementary Figure 3

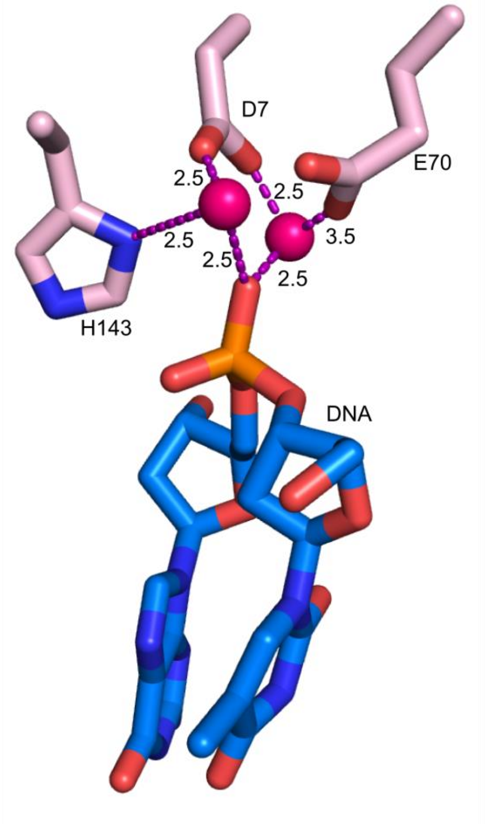

Supplementary Figure 3. Interatomic distance restraints (dashed lines) that were used to approximate the catalytically relevant geometry in simulations of the RuvC-DNA complex. Active site residues are shown as sticks and labeled. The DNA is shown in blue with scissile phosphate in orange (phosphorus) and red (oxygen). Magnesium ions are shown as pink spheres. The upper-bound (maximum) distances that are permitted by the individual restraints are given in Å. Interatomic distances beyond the specified limits were penalized with a linearly increasing energy that was defined by a force constant of 50 kcal × mol^{-1} × $Å^{-1}$.

Supplementary Figure 4

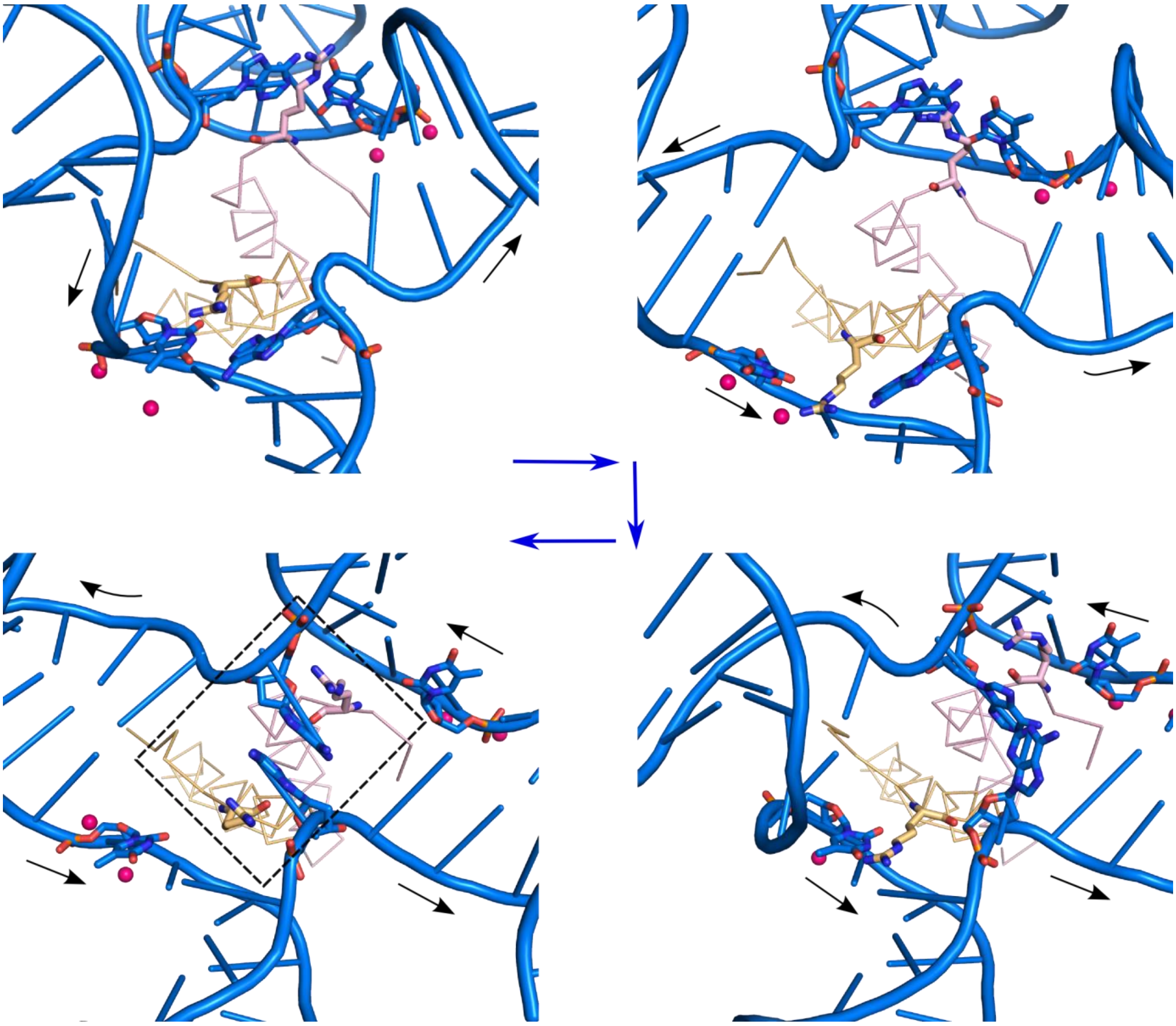

Supplementary Figure 4. Gradual disruption of the two scissile T-A base pairs and the subsequent formation of Arg76/adenine/adenine/Arg76 cross-junction stacking as suggested by REST2 simulations of the RuvC-DNA complex. The black arrows show 5' to 3' polarity of DNA strands, the blue arrows indicate time progression. The base pairs on both sides of the scissile phosphate and Arg76 side-chains are shown as sticks and the nearby backbone of the protein is shown as a wire representation. The stack is indicated with dashed box.

Supplementary Figure 5

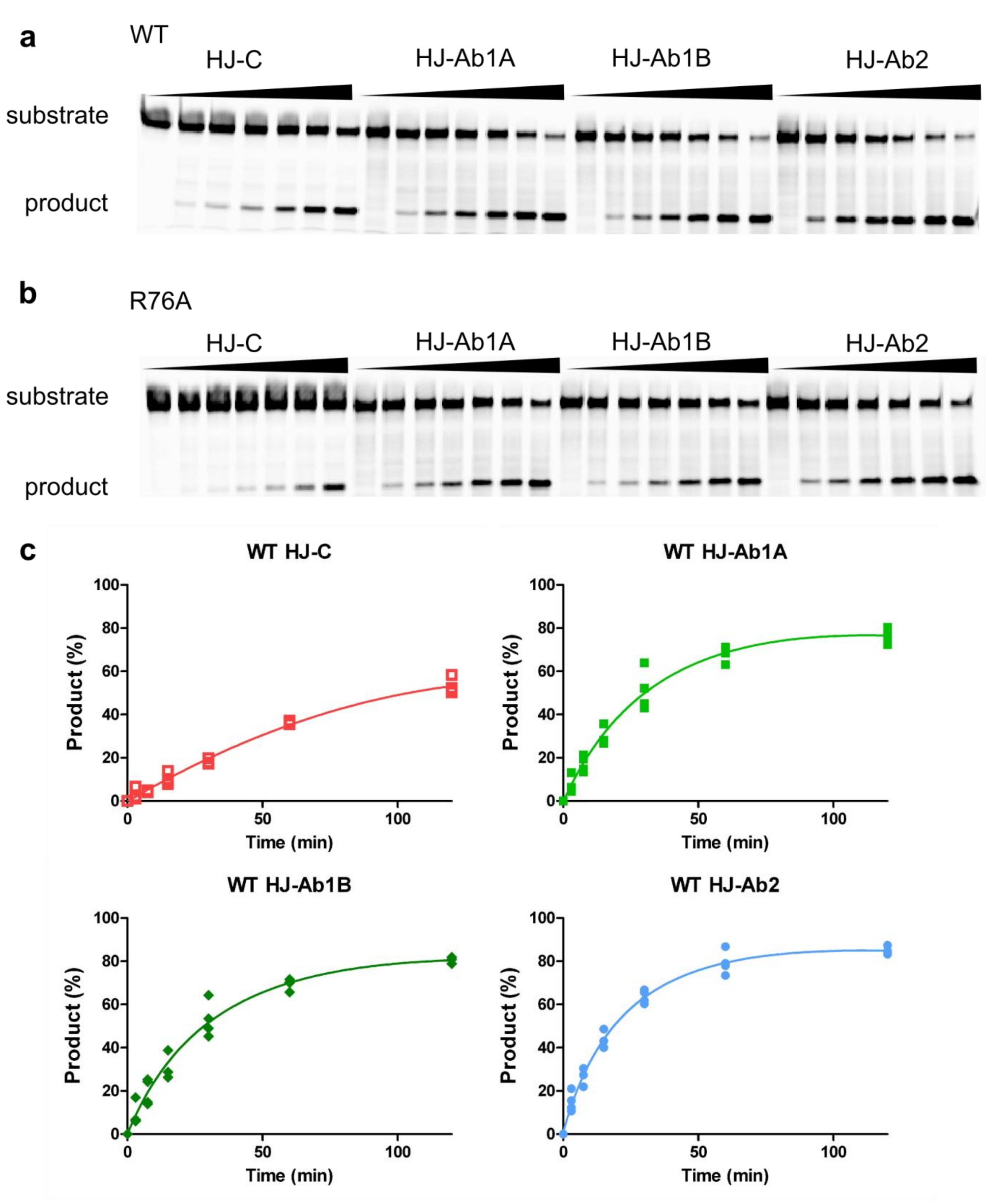

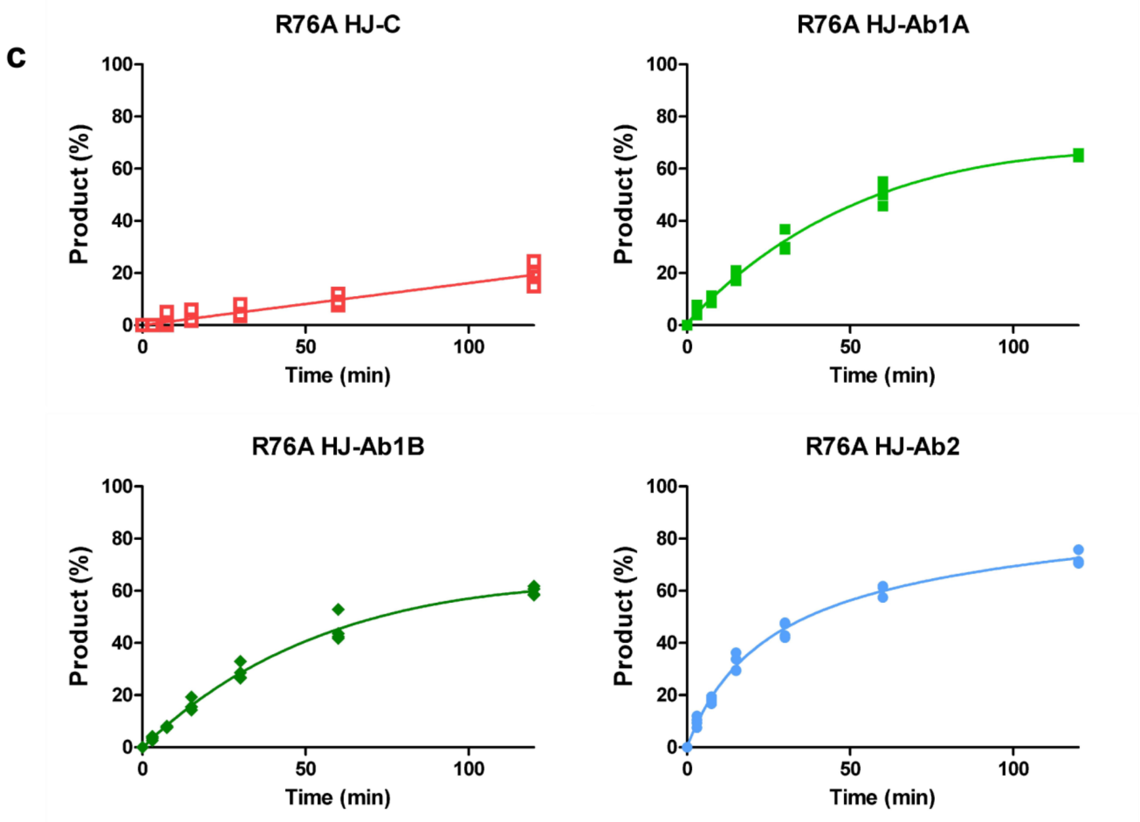

Supplementary Figure 5. Activity of *Tt*-RuvC with substrates with abasic sites. (**a, b**) Representative gels scanned for fluorescence. Holliday junction resolving activity of wildtype protein (a) and its R76A variant (b) was tested on substrates with fluorescently labeled cleaved strands. The protein was mixed with the substrate at a 2:1 molar ratio. The reaction products were resolved on 12% TBE-urea-formamide denaturing gels. The time range of the reaction (0-120 min) is indicated by a triangle. (**c**) Plots of the data after gel densitometry showing individual data points.

Supplementary Figure 6

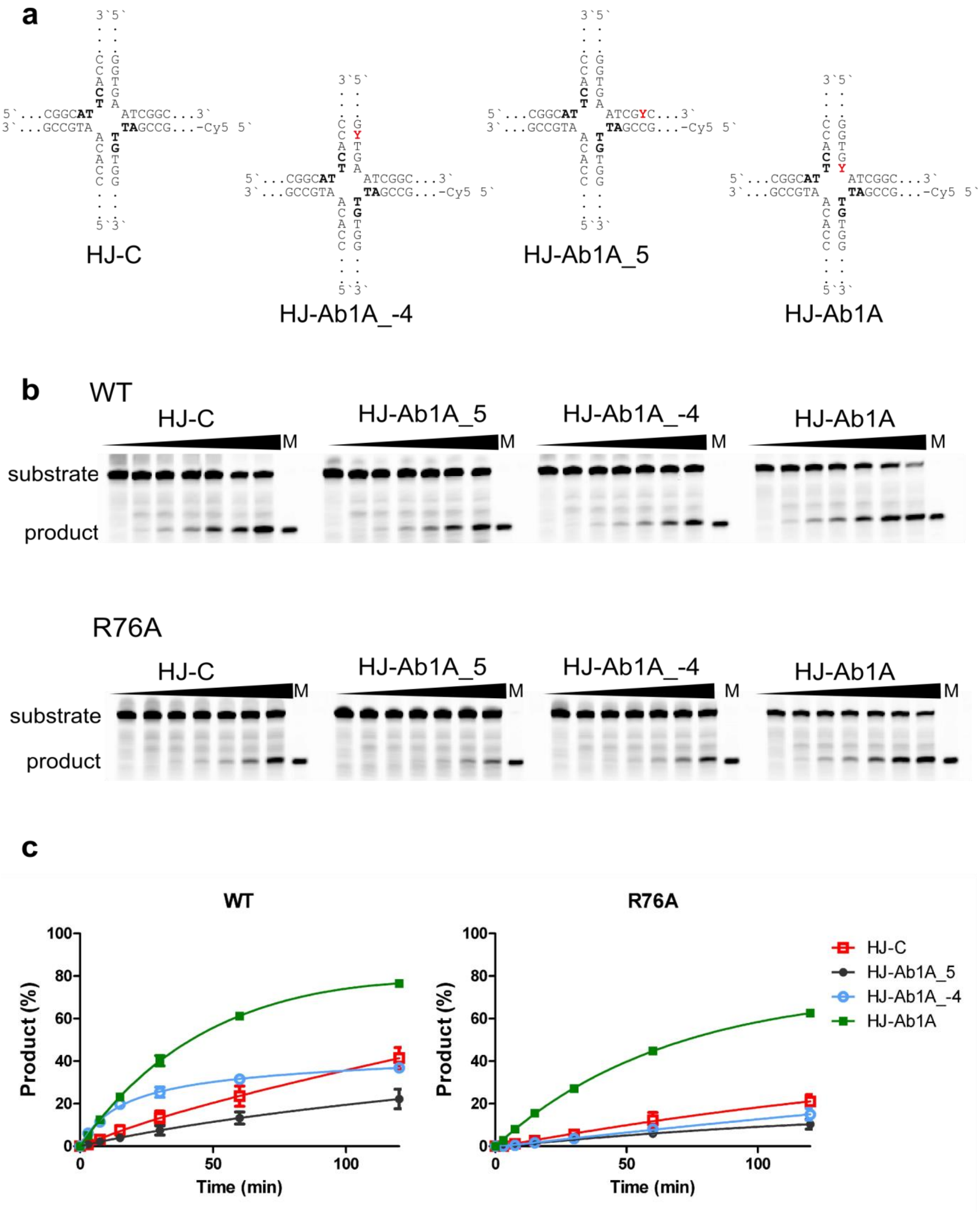

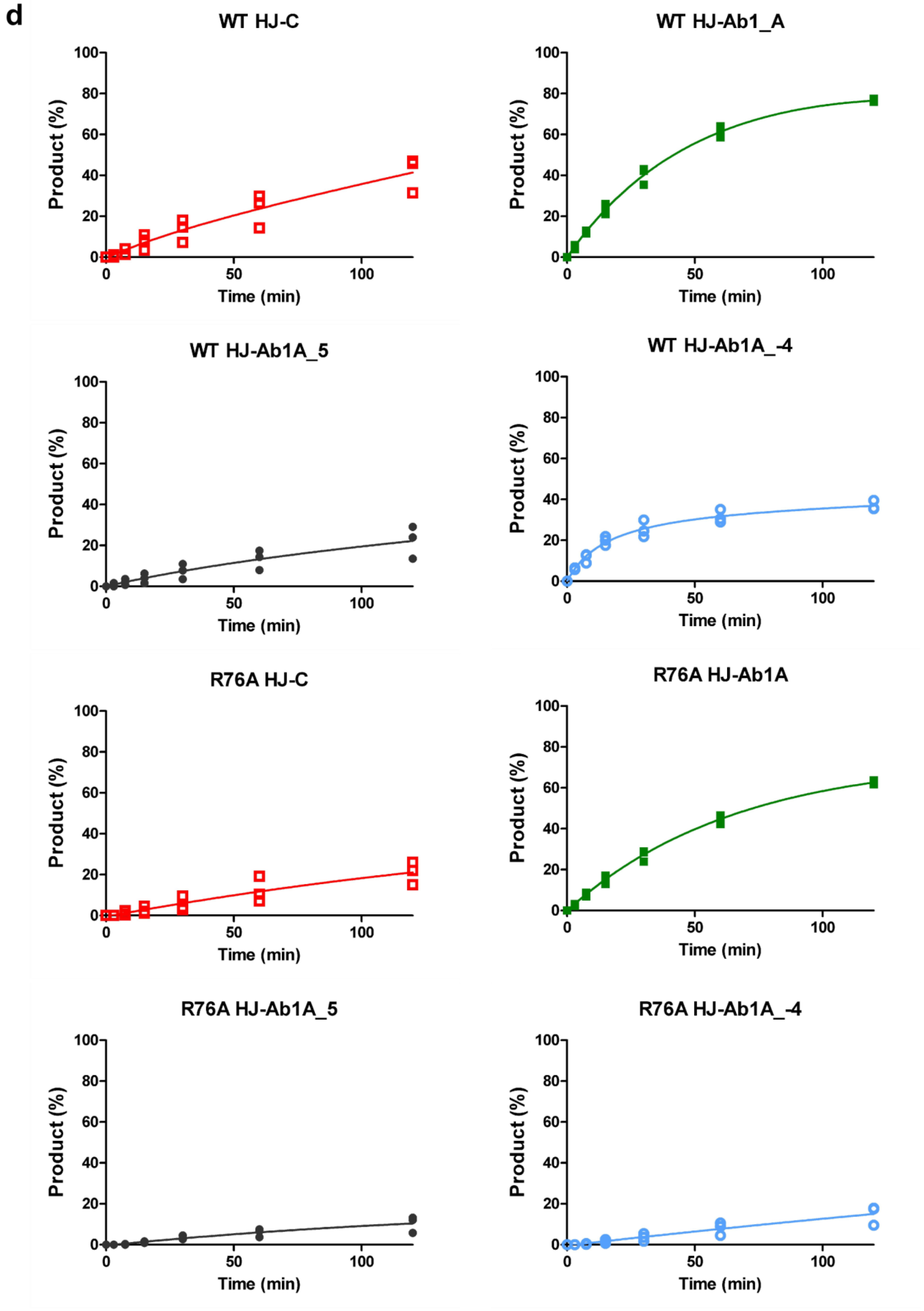

Supplementary Figure 6. Activity of *Tt*-RuvC and its R76A variant with control substrates with abasic sites. (**a**) Sequences of the substrates with abasic sites. (**b**) Scans of representative 12% TBE-

urea-formamide denaturing gels. Holliday junction resolving activity was tested on substrates with fluorescently labeled cleaved strands, and the reaction products were resolved on the gels which were scanned for fluorescence. M: reaction product marker. (**c**) Plots of the data after gel densitometry. Data from three independent experiments were averaged and plotted for each time-point. Error bars represent the standard deviation. (**d**) Plots of the data showing individual data points.

Supplementary Figure 7

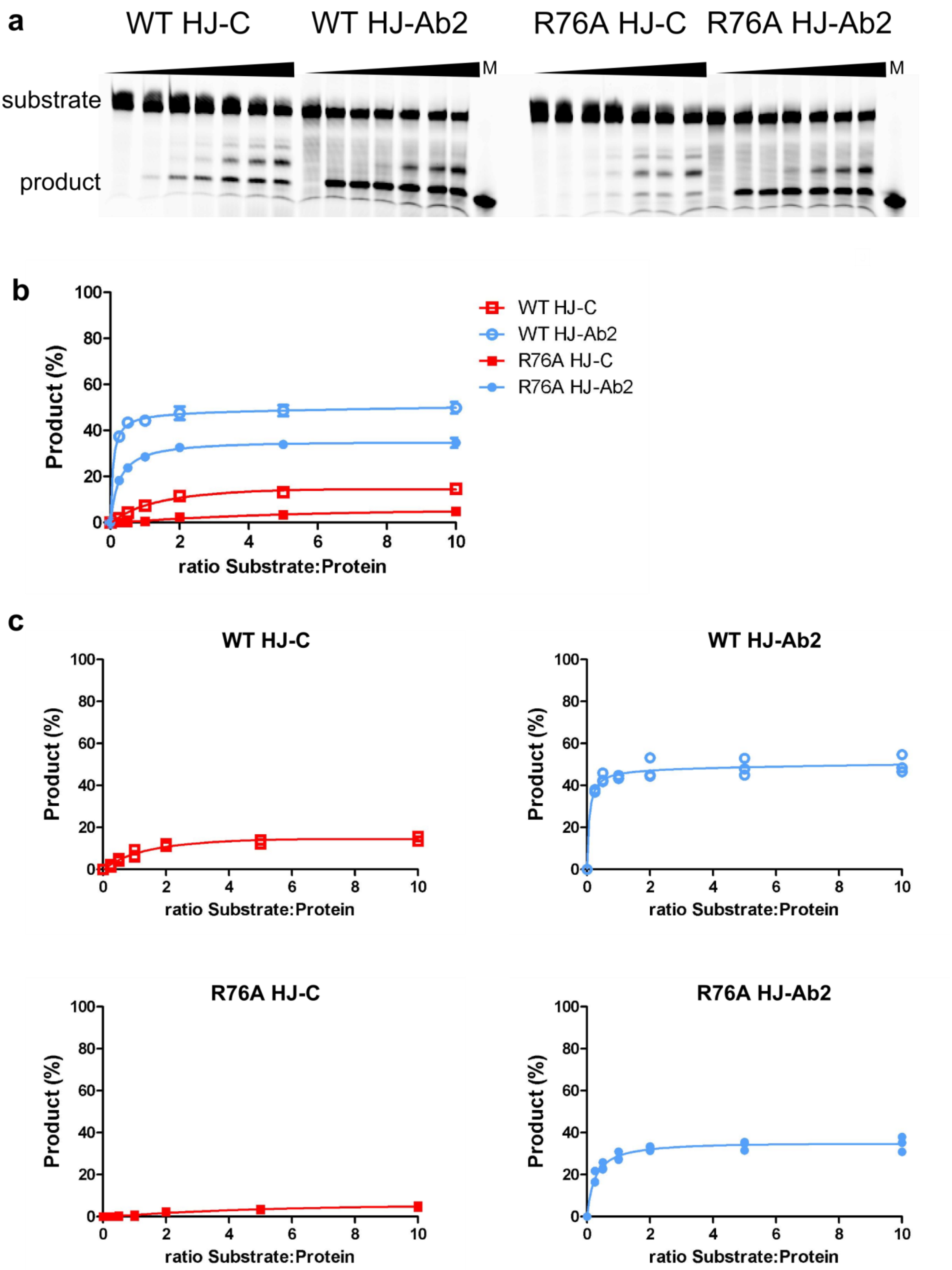

Supplementary Figure 7. Activity of *Tt*-RuvC and its R76A variant on HJ substrates (different substrate:protein ratios). Holliday junction resolving activity was tested on the control HJ and a

substrate with abasic sites (HJ-Ab2, see Fig 4c for DNA sequences). The cleaved DNA strands were fluorescently labeled, amount of oligo was fixed (25 nM). (**a**) Scans of representative 12% TBE-urea-formamide denaturing gels. Holliday junction resolving activity was tested at different substrate to protein molar ratios (1:0.25 to 1:10) and the reaction products were resolved on the gels which were scanned for fluorescence. M: reaction product marker. (**b**) Plots of the data after gel densitometry. Data from three independent experiments were averaged and plotted for each time-point. Error bars represent the standard deviation. (**c**) Plots of the data showing individual data points.

Supplementary Figure 8

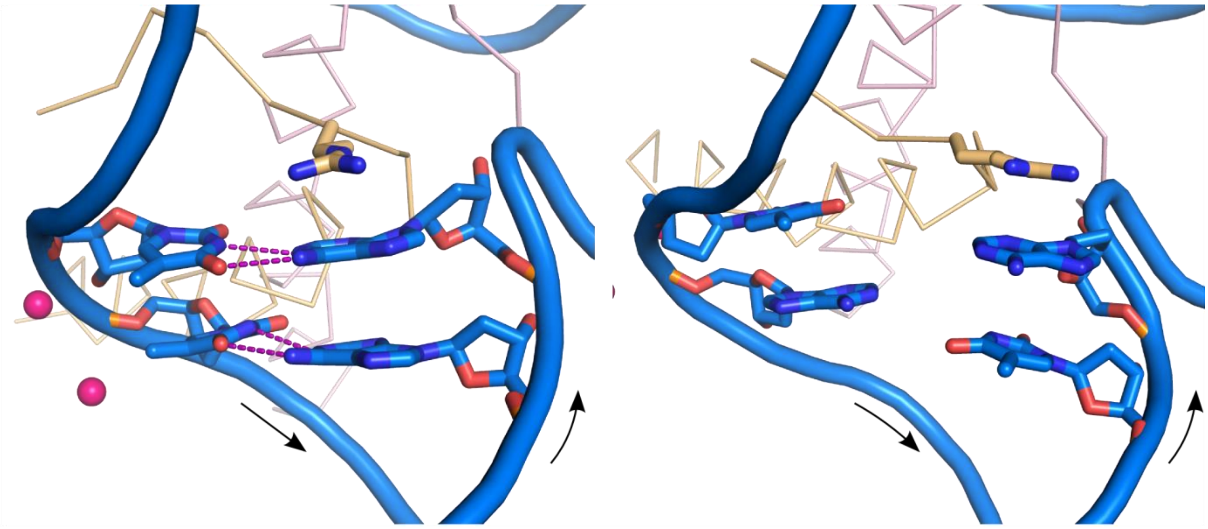

Supplementary Figure 8. Simultaneous disruption of the base pairs upstream and downstream to the scissile phosphate in simulations in which the C-G/G-C downstream base pairs were replaced with T-A/A-T. The base pairs on both sides of the scissile phosphate and Arg76 side-chain are shown as sticks and the nearby backbone of the protein is shown as a wire representation. The black arrows schow 5′ to 3′ polarity of DNA strands. The dashed purple lines indicate H-bonds. Left panel – properly formed base pairs, right panel – disrupted base pairs.

Supplementary Figure 9

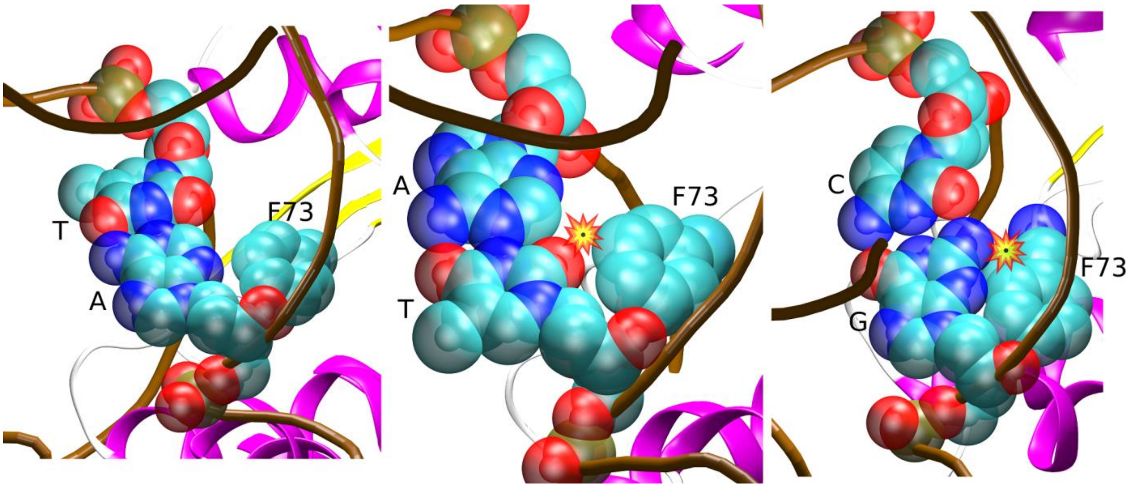

Supplementary Figure 9. Contacts of the Phe73 side-chain with the second base pair of the cleavage consensus. The size of the atoms is displayed according to their Van der Waals radii. The presence of bulky exocyclic groups causes steric clashes (explosion symbol) in contacts between the Phe73 side-chain and thymine (middle) or guanine (right).

Supplementary Figure 10

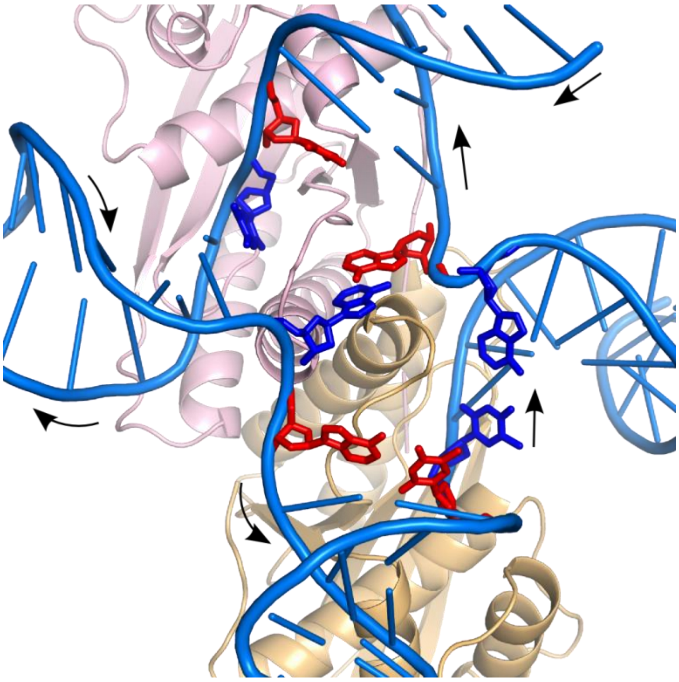

Supplementary Figure 10. Hypothetical model of cross-junction stacking between adenines from the scissile base pair (blue) and second base pair of the consensus sequence (red). The black arrows show 5' to 3' polarity of DNA strands.

Supplementary Figure 11

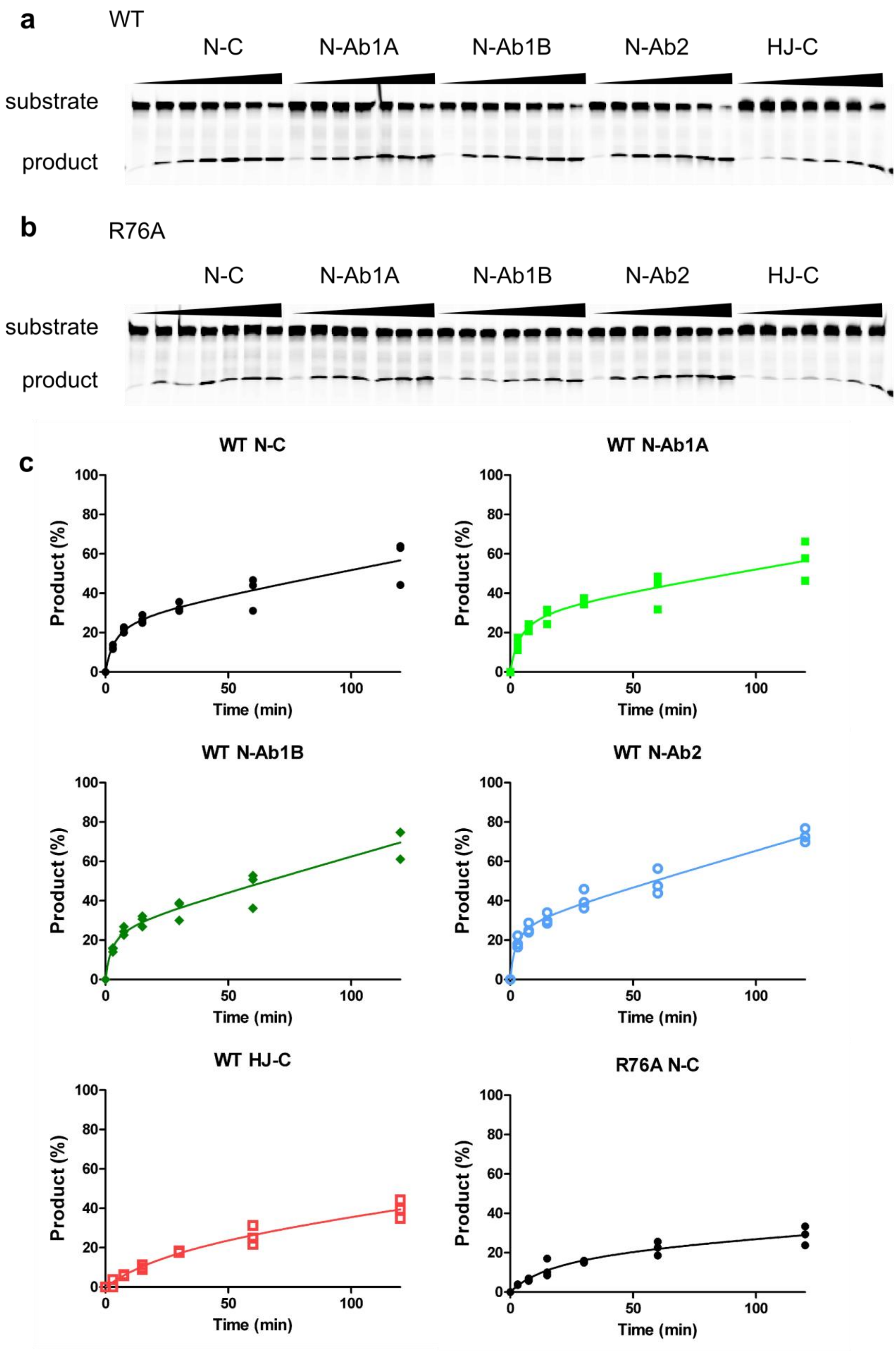

c

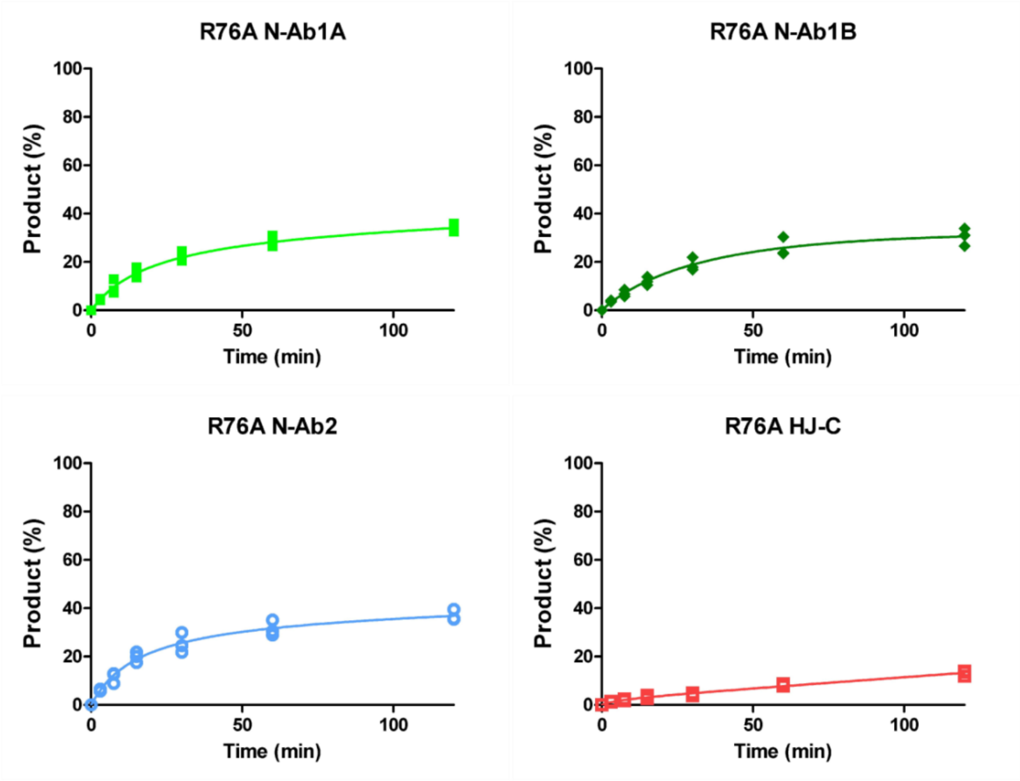

Supplementary Figure 11. Activity of *Tt*-RuvC and its R76A variant with nicked substrates. (**a, b**) Representative gel scanned for fluorescence. Holliday junction resolving activity of wildtype protein (a) and its R76A variant (b) was tested on substrates with fluorescently labeled cleaved strands. The protein was mixed with the substrate at a 2:1 molar ratio. The reaction products were resolved on 12% TBE-urea-formamide denaturing gels. The time range of the reaction (0-120 min) is indicated by a triangle. (**c**) Plots of the data after gel densitometry showing individual data points.

Supplementary Figure 12

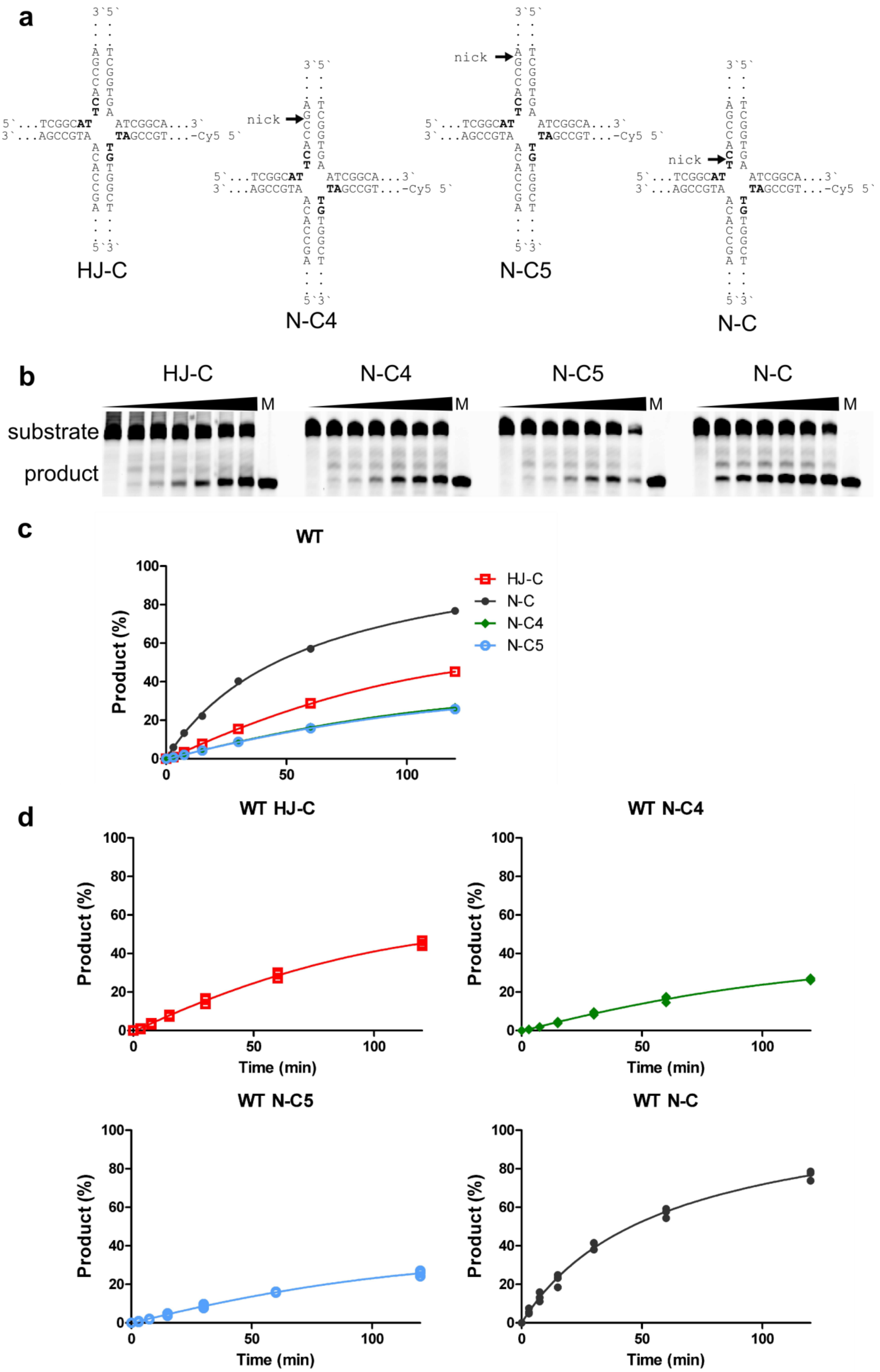

Supplementary Figure 12. Activity of *Tt*-RuvC with control substrates with nicks in cleaved strands. (**a**) Sequences of the substrates with nicks. (**b**) Fluorescence scans of representative 12% TBE-urea-formamide denaturing gels. Holliday junction resolving activity was tested on substrates with fluorescently labeled cleaved strands, and the reaction products were resolved on gels. The protein was mixed with the substrate at a 2:1 molar ratio. The time range of the reaction (0-120 min) is indicated by a triangle. M: reaction product marker. (**c**) Plots of the data after gel densitometry. Data from three independent experiments were averaged and plotted for each time-point. Error bars represent the standard deviation. (**d**) Plots of the data showing individual data points.

Supplementary Figure 13

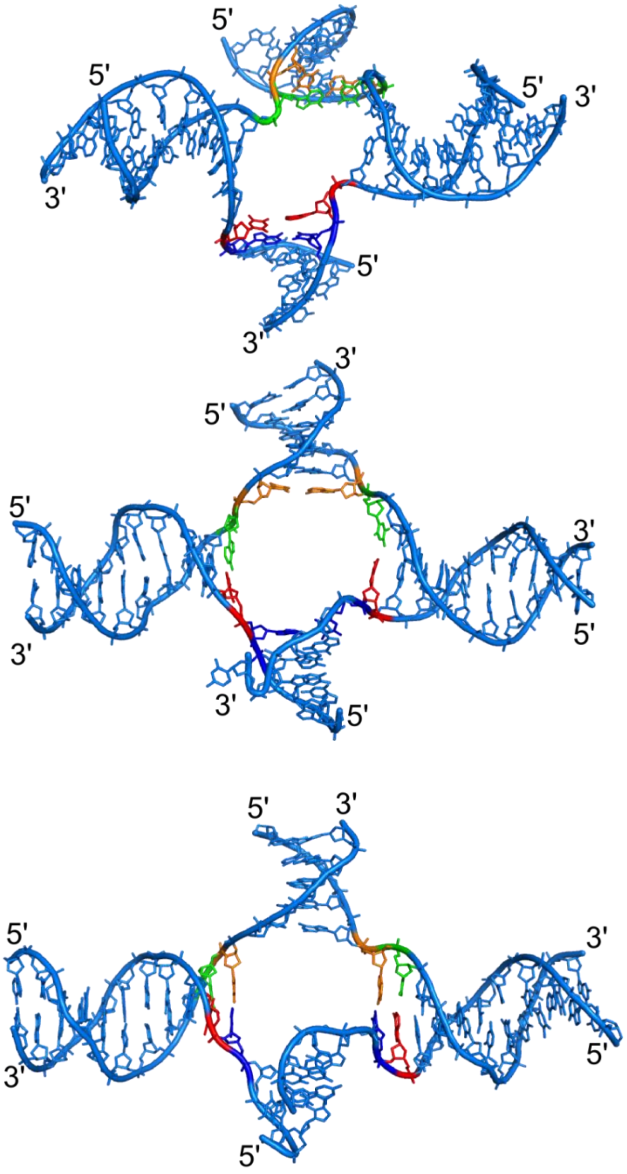

Supplementary Figure 13. Extensive junction dynamics in MD simulation of free HJ DNA. The left panel shows the starting structure. The middle and right panels show later points in the simulation. The base pairs that undergo changes in base pairing as a result of branch point migration are shown in green, red, orange, and dark blue.

Supplementary Table 1. Data collection and refinement statistics.

**Data collection**	**RuvC-HJ complex**
Space group	$P\ 3_2\ 2\ 1$
Cell dimensions	
a, b, c (Å)	108.07, 108.07, 133.3
α, β, γ (°)	90, 90, 120
Resolution (Å)	38.3-3.4 (3.5-3.4)
$CC_{1/2}$	100 (71.7)
I / σI	16.8 (1.8)
Completeness (%)	99.5 (98.7)
Redundancy	5.6 (5.6)
**Refinement**	
Resolution (Å)	38.3-3.4
No. reflections	12660
R_{work} / R_{free} (%)	18.1/23.1
No. atoms	3479
Protein	2325
Nucleic acid/ion	1149
B-factors (Å^2)	
Protein	111.4
Nucleic acid/ion	142.3
RMSD	
Bond lengths (Å)	0.01
Bond angles (°)	1.17

Supplementary Table 2. List of simulations.

Standard MD	
**Simulated system**[a]	**Number of simulations × length (ns)**
protein/DNA	1 × 500
protein/DNA, cognate, Mg^{2+}	1 × 500
protein/DNA, cognate, Mg^{2+}, rst	1 × 500
protein/DNA, cognate, Mg^{2+}, scissile TA→AT	1 × 1000, 2 × 500
protein/DNA, cognate, Mg^{2+}, rst, scissile TA→AT	2 × 500
protein/DNA, cognate, Mg^{2+}, preceding TA→AT	1 × 200
protein/DNA, cognate, Mg^{2+}, preceding TA→CG	1 × 200
protein/DNA, cognate, Mg^{2+}, succeeding CG→TA	2 × 1000
protein/DNA, cognate, Mg^{2+}, rst, R76A	1 × 1000
protein/DNA, cognate, Mg^{2+}, cross-junction[b]	1 × 2000
protein/DNA, cognate, Mg^{2+}, nicked	2 × 1000, 2 × 500
protein/DNA, half-cognate, Mg^{2+}, nicked	1 × 1000
DNA, cognate	1 × 500
**REST2 simulations**	
**Simulated system**	**Number of replicas × length (ns)**
protein/DNA, cognate	8 × 3000
protein/DNA, cognate, Mg^{2+}	8 × 5000
protein/DNA, cognate, Mg^{2+}, rst	8 × 2000
protein/DNA, cognate, Mg^{2+}, scissile TA→AT	8 × 2000
protein/DNA, cognate, Mg^{2+}, preceding TA→AT	8 × 2000

[a]**Cognate** – the DNA sequence of the crystal structure was modified to agree with the consensus (A/TTT↓C/G) DNA sequence that is preferentially cleaved by RuvC. The downwards arrow marks the point of cleavage. **Mg^{2+}** – two magnesium ions were bound at both catalytic centers (total of four magnesium ions per system). **Rst** – interatomic distance restraints were applied to promote the

approximate catalytic geometry in both catalytic centers (see Methods and Fig S3.). **Scissile, preceding, succeeding –** the ***scissile*** T-A base pair, T-A base pair that ***precedes*** the scissile base pair, or the C-G/G-C base pair that ***succeeds*** the scissile base pair, respectively, were mutated. **R76A –** the specified amino-acid residue of RuvC was substituted with alanine. **Nicked** – the backbone of the DNA substrate was already cleaved (nicked) at the first catalytic site. **Half-cognate** – the DNA sequence contained the cognate sequence that is preferentially cleaved by RuvC only at the first catalytic site, whereas the non-cognate sequence was located at the second site.

[b]The standard MD simulation began from a snapshot of the (protein/DNA, cognate, Mg^{2+}) REST2 simulation that contained the Arg76/adenine/adenine/Arg76 cross-junction stack.

Supplementary Table 3. Affinity of RuvC (wild type and R76A variant) for HJ substrates, measured by fluorescence anisotropy.

	K_d (μM)	SD
WT+HJ-C	0.84	0.30
WT+HJ-Ab1A	0.51	0.13
WT+HJ-Ab1B	1.24	0.29
WT+HJ-Ab2	0.75	0.19
WT+N-C	1.00	0.18
WT+N-Ab1A	1.77	0.41
WT+N-Ab1B	2.27	0.63
WT+N-Ab2	1.35	0.30
R76A+HJ-C	3.75	1.99
R76A+HJ-Ab1A	1.18	0.25
R76A+HJ-Ab1B	2.43	1.38
R76A+HJ-Ab2	1.03	0.20

SD, standard deviation.

Supplementary Table 4. Oligonucleotide sequences.

Oligonucleotides	
Name	Sequence
J221	5'-CAATCGGCTTTGACCTTTGGTCAATCGGCAGAT-3'
J222	5'-ATCTGCCGATTCTGGTTTCCAGAAAGCCGATTG-3'
HJ-1 HEX	5'HEX-GGTAGGACGGCCTCGCAATCGGCATTCACCGAGCACGCGAGATGTCAACG-3'
HJ-2 Ab	5'-CGTTGACATCTCGCGTGCTCGGTGYATCGGCAGATGCGGAGTGAAGTTCC-3'
HJ-2 Ab_-4	5'-CGTTGACATCTCGCGTGCTCGYTGAATCGGCAGATGCGGAGTGAAGTTCC-3'
HJ-2 Ab_5	5'-CGTTGACATCTCGCGTGCTCGGTGAATCGYCAGATGCGGAGTGAAGTTCC-3'
HJ-3 Cy5	5'Cy5-GGAACTTCACTCCGCATCTGCCGATTGTGGCTGTGGCGTGTTTCTGGTGG-3'
HJ-4 Ab	5'-CCACCAGAAACACGCCACAGCCACYATGCCGATTGCGAGGCCGTCCTACC-3'
HJ-2	5'-CGTTGACATCTCGCGTGCTCGGTGAATCGGCAGATGCGGAGTGAAGTTCC-3'
HJ-4	5'-CCACCAGAAACACGCCACAGCCACAATGCCGATTGCGAGGCCGTCCTACC-3'
Nick A	5'-GGTAGGACGGCCTCGCAATCGGCATT-3'
Nick B	5'-phosphate-CACCGAGCACGCGAGATGTCAACG-3'
Nick A4	5'- GGTAGGACGGCCTCGCAATCGGCATTCACC – 3'
Nick B4	5'-phosphate-GAGCACGCGAGATGTCAACG -3'
Nick A5	5'- GGTAGGACGGCCTCGCAATCGGCATTCACCG – 3'
Nick B5	5'-phosphate-AGCACGCGAGATGTCAACG -3'
HJ-1 ap	5'-GGTAGGACGGCCTCGCAATCGGCATTGACCGAGCACGCGAGATGTCAACG-3'
HJ-2 AP 1	5'-CGTTGACATCTCGCGTGCTCGGTCXGCAGGCAGATGCGGAGTGAAGTTCC-3'
HJ-3 ap	5'-GGAACTTCACTCCGCATCTGCCTGCGCTGGCTGTGGCGTGTTTCTGGTGG-3'
HJ-4 ap	5'-CCACCAGAAACACGCCACAGCCAGCATGCCGATTGCGAGGCCGTCCTACC-3'
HJ-2 AP2	5'-CGTTGACATCTCGCGTGCTCGGTCAGCXGGCAGATGCGGAGTGAAGTTCC-3'
Substrates	
Name	Oligonucleotides
J221/J222	J221, J222
AP1	HJ-1 ap, HJ-2 AP 1, HJ-3 ap, HJ-4 ap
AP2	HJ-1 ap, HJ-2 AP2, HJ-3 ap, HJ-4 ap

HJ-C	HJ-1 HEX, HJ-2, HJ-3 Cy5, HJ-4
HJ-Ab1A	HJ-1 HEX, HJ-2 Ab, HJ-3 Cy5, HJ-4
HJ-Ab1B	HJ-1 HEX, HJ-2, HJ-3 Cy5, HJ-4 Ab
HJ-Ab2	HJ-1 HEX, HJ-2 Ab, HJ-3 Cy5, HJ-4 Ab
N-C	Nick A, Nick B, HJ-2, HJ-3 Cy5, HJ-4
N-Ab1A	Nick A, Nick B, HJ-2 Ab, HJ-3 Cy5, HJ-4
N-Ab1B	Nick A, Nick B, HJ-2 , HJ-3 Cy5, HJ-4 Ab
N-Ab2	Nick A, Nick B, HJ-2 Ab, HJ-3 Cy5, HJ-4 Ab
N-C4	Nick A4, Nick B4, HJ-2, HJ-3 Cy5, HJ-4
N-C5	Nick A5, Nick B5, HJ-2, HJ-3 Cy5, HJ-4
HJ-Ab1A_-4	HJ-1 HEX, HJ-2 Ab_-4, HJ-3 Cy5, HJ-4
HJ-Ab1A_5	HJ-1 HEX, HJ-2 Ab_5, HJ-3 Cy5, HJ-4

X = 2-aminopurine, Y = abasic site

SUPPLEMENTARY REFERENCES

1 Maier, J. A. *et al.* ff14SB: Improving the Accuracy of Protein Side Chain and Backbone Parameters from ff99SB. *Journal of chemical theory and computation* **11**, 3696-3713, (2015).

2 Zgarbova, M. *et al.* Refinement of the Sugar-Phosphate Backbone Torsion Beta for AMBER Force Fields Improves the Description of Z- and B-DNA. *Journal of chemical theory and computation* **11**, 5723-5736, (2015).

3 Cornell, W. D. *et al.* A Second Generation Force Field for the Simulation of Proteins, Nucleic Acids, and Organic Molecules. *Journal of the American Chemical Society* **117**, 5179-5197, (1995).

4 Hornak, V. *et al.* Comparison of multiple Amber force fields and development of improved protein backbone parameters. *Proteins* **65**, 712-725, (2006).

5 Sponer, J. *et al.* How to understand atomistic molecular dynamics simulations of RNA and protein-RNA complexes? *Wiley interdisciplinary reviews. RNA* **8**, (2017).

6 Sponer, J. *et al.* RNA Structural Dynamics As Captured by Molecular Simulations: A Comprehensive Overview. *Chemical reviews* **118**, 4177-4338, (2018).

7 Perez, A. *et al.* Refinement of the AMBER force field for nucleic acids: improving the description of alpha/gamma conformers. *Biophysical journal* **92**, 3817-3829, (2007).

8 Krepl, M. *et al.* Reference simulations of noncanonical nucleic acids with different chi variants of the AMBER force field: quadruplex DNA, quadruplex RNA and Z-DNA. *Journal of chemical theory and computation* **8**, 2506-2520, (2012).

9 Zgarbova, M. *et al.* Toward Improved Description of DNA Backbone: Revisiting Epsilon and Zeta Torsion Force Field Parameters. *Journal of chemical theory and computation* **9**, 2339-2354, (2013).

10 Krepl, M. *et al.* Can We Execute Stable Microsecond-Scale Atomistic Simulations of Protein-RNA Complexes? *Journal of chemical theory and computation* **11**, 1220-1243, (2015).

11 Krepl, M. *et al.* An intricate balance of hydrogen bonding, ion atmosphere and dynamics facilitates a seamless uracil to cytosine substitution in the U-turn of the neomycin-sensing riboswitch. *Nucleic acids research* **46**, 6528-6543, (2018).

12 Needham, P. J., Bhuiyan, A. & Walker, R. C. Extension of the AMBER molecular dynamics software to Intel's Many Integrated Core (MIC) architecture. *Comput. Phys. Commun.* **201**, 95-105, (2016).

13 Darden, T., York, D. & Pedersen, L. Particle mesh Ewald: An N·log(N) method for Ewald sums in large systems. **98**, 10089-10092, (1993).

14 Zuo, Z. & Liu, J. Cas9-catalyzed DNA Cleavage Generates Staggered Ends: Evidence from Molecular Dynamics Simulations. *Scientific reports* **5**, 37584, (2016).

15 Krasovska, M. V. *et al.* Cations and hydration in catalytic RNA: molecular dynamics of the hepatitis delta virus ribozyme. *Biophysical journal* **91**, 626-638, (2006).

16 Ke, A., Ding, F., Batchelor, J. D. & Doudna, J. A. Structural roles of monovalent cations in the HDV ribozyme. *Structure (London, England : 1993)* **15**, 281-287, (2007).

17 Nowotny, M. *et al.* Structure of human RNase H1 complexed with an RNA/DNA hybrid: insight into HIV reverse transcription. *Molecular cell* **28**, 264-276, (2007).

18 Chen, L., Shi, K., Yin, Z. & Aihara, H. Structural asymmetry in the Thermus thermophilus RuvC dimer suggests a basis for sequential strand cleavages during Holliday junction resolution. *Nucleic acids research* **41**, 648-656, (2013).

19 Bennett, R. J., Dunderdale, H. J. & West, S. C. Resolution of Holliday junctions by RuvC resolvase: cleavage specificity and DNA distortion. *Cell* **74**, 1021-1031, (1993).

20 Fogg, J. M., Schofield, M. J., White, M. F. & Lilley, D. M. Sequence and functional-group specificity for cleavage of DNA junctions by RuvC of Escherichia coli. *Biochemistry* **38**, 11349-11358, (1999).

21 Shah, R., Bennett, R. J. & West, S. C. Genetic recombination in E. coli: RuvC protein cleaves Holliday junctions at resolution hotspots in vitro. *Cell* **79**, 853-864, (1994).

22 Ichiyanagi, K., Iwasaki, H., Hishida, T. & Shinagawa, H. Mutational analysis on structure-function relationship of a holliday junction specific endonuclease RuvC. *Genes to cells : devoted to molecular & cellular mechanisms* **3**, 575-586, (1998).

23 Yoshikawa, M., Iwasaki, H. & Shinagawa, H. Evidence that phenylalanine 69 in Escherichia coli RuvC resolvase forms a stacking interaction during binding and destabilization of a Holliday junction DNA substrate. *The Journal of biological chemistry* **276**, 10432-10436, (2001).

24 Wheatley, E. G., Pieniazek, S. N., Mukerji, I. & Beveridge, D. L. Molecular dynamics of a DNA Holliday junction: the inverted repeat sequence d(CCGGTACCGG)(4). *Biophysical journal* **102**, 552-560, (2012).

25 Yadav, R. K. & Yadava, U. Molecular dynamics simulation of hydrated d(CGGGTACCCG)4 as a four-way DNA Holliday junction and comparison with the crystallographic structure. *Molecular Simulation* **42**, 25-30, (2016).

26 Yoo, J. & Aksimentiev, A. New tricks for old dogs: improving the accuracy of biomolecular force fields by pair-specific corrections to non-bonded interactions. *Physical chemistry chemical physics : PCCP* **20**, 8432-8449, (2018).

27 Yu, J., Ha, T. & Schulten, K. Conformational model of the Holliday junction transition deduced from molecular dynamics simulations. *Nucleic acids research* **32**, 6683-6695, (2004).

28 Stadlbauer, P., Krepl, M., Cheatham, T. E., 3rd, Koca, J. & Sponer, J. Structural dynamics of possible late-stage intermediates in folding of quadruplex DNA studied by molecular simulations. *Nucleic acids research* **41**, 7128-7143, (2013).
