# Supplementary material for: RuvC uses dynamic probing of the Holliday junction to achieve sequence specificity and efficient resolution
Source: Nat Commun. 2019 Sep 10;10:4102. doi: 10.1038/s41467-019-11900-8 (PMC6736871; doi:10.1038/s41467-019-11900-8)
Supplement: Supplementary file 3 — Description of Additional Supplementary Files [file 41467_2019_11900_MOESM3_ESM.pdf]

Description of Additional Supplementary Files

File Name: Supplementary Movie 1

Description: **Conformational changes at the exchange point of the HJ** (example of Molecular Dynamics results, the frames show every 250th model from simulations). Arg76 is shown in sticks and the protein backbone of the loop harboring it in pink cartoon. The DNA is shown in blue ladder with the bases at the exchange point in darker color and in stick representation. The scissile phosphate is shown as a sphere.
